# Opinion on the event-related potential signature of automatic detection of violated regularity (visual mismatch negativity): non-perceptual but predictive

**DOI:** 10.3389/fnhum.2023.1295431

**Published:** 2023-11-15

**Authors:** István Czigler

**Affiliations:** Institute of Cognitive Neuroscience and Psychology, Research Center of Natural Sciences, Budapest, Hungary

**Keywords:** visual mismatch negativity, post-perceptual, predictive coding, automatic change detection, processing duration

## Introduction

I argue that the electrophysiological phenomenon, the visual mismatch negativity (vMMN), considered to be the signature of automatic visual change detection is post-perceptual.^1^ In other words, it emerges after the identification of the eliciting events. My argument is that simple, visual processes leading to stimulus identification are faster than the time range of electrophysiological phenomenon, the visual mismatch negativity component of event-related potentials (ERPs).

Presenting stimulus sequences with frequent physically or categorically identical visual stimuli (standards) and rare stimuli (deviant) that violate the regularity of standards, an ERP component, the vMMN emerges, even if these stimuli are unrelated to the ongoing task (passive oddball paradigm). VMMN is observed in the ERP when stimulus sequences with frequent physically or categorically identical visual stimuli (standards) and rare stimuli that violate the regularity of the standards (deviants) are presented. It is considered the counterpart of the auditory mismatch negativity (for reviews see Stefanics et al., 2014 and Garrido et al., 2009, for the visual and auditory MMN, respectively). Sources of this component have been identified in various regions of the visual cortex as well as, in some cases, in the frontal cortex (e.g. Kimura et al., 2010). The vMMN latency range is between 120 and 350 ms post-stimulus. VMMN is elicited by events unrelated to the ongoing task, outside the location of the task, and emerges to stimuli outside conscious awareness (Berti, 2011; Jock et al., 2017), therefore it is considered to be a result of automatic processes.

VMMN can be elicited by deviant visual features such as color (Athanasopoulos et al., 2010), spatial frequency (Heslenfeld, 2003), movement direction (Lorenzo-López et al., 2004), and orientation (Kimura et al., 2009). VMMN is also elicited by higher-order deviant visual characteristics, like object-related deviancy (Müller et al., 2010), facial attributes (Astikainen and Hietanen, 2009; Kecskés-Kovács et al., 2013; Kreegipuu et al., 2013), and semantic categories (Hu et al., 2020).

The dominant account of the function of processes underlying (auditory) MMN and vMMN is the predictive coding theory. In summary, the mechanism underlying (auditory) MMN), and *mutatis mutandis*, vMMN, as Garrido et al. (2009) clearly state, are brain activity within perception: “Predictive coding (or, more generally, hierarchical inference in the brain) states that perception arises from integrating sensory information from the environment and our prediction based on a model of what caused that sensory information” (p. 459). Accordingly, vMMN is a signature of a cascade (hierarchy) of comparison processes between the content of the representation of the actual input events and a memory store of expected events. The hierarchical predictive coding framework claims that veridical perception is supported by neural processes optimizing probabilistic representations of the causes of sensory inputs (Friston, 2010). In the vMMN studies the probabilistic representation is based on the high probability of the standard. The cascade terminates when the updated memory content matches the input representation. This account claims that vMMN depends on the characteristics of the memory content established by the regular event sequences. Presenting an expected event (a standard stimulus in the oddball paradigm) does not elicit vMMN, but a change in the event sequence (a deviant stimulus) initiates this cascade-type activity underlying vMMN. VMMN is an “error signal,” and the function of the “vMMN-generating system” is to update our predictive model of the world through prediction errors and infer the likely causes of the sensory inputs. According to this account, vMMN is a signature of activity within the perceptual system, and the processes underlying vMMN serve the identification of deviant events.

## The time range of VMMN

VMMN is usually measured on the difference potentials, where ERPs to the standard are subtracted from the ERPs to deviant.^2^ Deviant minus standard differences may appear in an earlier (120–200 ms) and a later (200–300/350 ms) range (e.g., Maekawa et al., 2005; File et al., 2017). The earlier latency range corresponds to the N1/N170 ERP component, and this deviant-standard difference is frequently attributed to the stimulus-specific adaptation (repetition suppression) of this component. On the other hand, the later component, sometimes called “genuine vMMN” is considered to be a consequence of additional processes, attributed to the cascade processes of the predictive coding account. Equal probability control (EQ) is used to separate the two effects. Within the EQ sequence, stimuli physically identical to those in the oddball sequences are embedded in a sequence with stimuli varying in the same parameter (e.g., orientation, but different values). The probability of each stimulus type is equal to the probability of the oddball deviant. The ERPs elicited by the oddball deviant and the physically identical EQ stimuli are compared afterward (deviant minus control difference potential; Jacobsen and Schroger, 2001). In fact, in some studies, this control eliminated the earlier part of the deviant minus standard difference (e.g., Kimura et al., 2009), but in other studies, this procedure resulted in an early “genuine vMMN” (e.g., for grating pattern Susac et al., 2014; for windmill pattern File et al., 2017, and also Kovarski et al., 2017; for dot patterns, Beck et al., 2021; for human faces Li et al., 2012).

At the later time range, deviant minus standard ERP differences appear after 250 ms, e.g., for dot motion (Rowe et al., 2020), facial emotions (e.g., Xiong et al., 2022), spatial frequency-filtered faces (Lacroix et al., 2022), spatial frequency (Tales et al., 2008), color (Sultson et al., 2019), line orientation (Kimura et al., 2009, but see Male et al., 2020).

In summary, vMMN emerges no earlier than 120 ms; and frequently terminates later than 300 ms. I claim that this range (i.e. including the earlier vMMN range) is later than the time needed to identify images depicting single objects, objects within contexts, and scenes.

## Data from reaction time for identifying visual events

The most direct method to measure the time needed to identify (plus decide and respond) visual events is a “go/no-go categorization task,” first introduced by Thorpe et al. (1996). Participants performed “animal/non-animal categorization” in reaction time (RT) situations, with concomitant EEG recording. Unmasked pictures of natural scenes were presented for 20 ms. The earliest behavioral responses were shorter than 300 ms. Interestingly, the onset of differential ERP activity in the two categories started 150 ms after stimulus onset. The research group developed a measure they termed minRT as the first-time bin of 10 ms, in which correct responses start to significantly outnumber incorrect responses (Fabre-Thorpe et al., 1998; VanRullen and Thorpe, 2001). MinRT for various categories (vehicles, animals) and in many variations of the paradigm (e.g., Rousselet et al., 2002; Macé et al., 2005; Joubert et al., 2007) was ~260 ms. Importantly, beyond the stimulus processing, this duration involves the duration of the decision process, motor organization, and response execution. Furthermore, in another response mode, when saccadic eye movement to the target category (natural scenes with or without animal) was measured instead of hand response, MinRT was 120 ms (Kirchner and Thorpe, 2006).

## Data from short-term conceptual memory

Potter and her colleagues introduced a series of studies presenting pictures using the rapid serial visual presentation (RSVP) method. In her now-classic study (Potter, 1976) she presented sequences of pictures with short exposure duration (the lowest being 113 ms), and with an inter-stimulus interval of 0 ms. A picture was a target when it contained a scene described before presenting the sequence. Detection of the target pictures was better than chance, even at 113 ms. Note that the subsequent pictures masked the previous ones, therefore processing time was restricted to the exposure duration. In a later study, Hagmann and Potter (2016) obtained an even shorter exposure time. In sequences of six pictures, and target definition after the sequence (i.e., without the possible involvement of attentional blink after the target (see Martens and Wyble, 2010 for a review), performance above chance level was observed for color, greyscale, and blurred pictures below 80 ms. It should be noted that pictures with only low spatial frequencies were not significantly detected at 80 ms exposure, showing that a presumed gist provided by the fast-conducting magnocellular system (e.g., Bar, 2009) was insufficient for the identification of the pictures. Concerning vMMN studies, in the case of oddball deviants like facial emotions (Kreegipuu et al., 2013), gender (Kecskés-Kovács et al., 2013), or right vs. left hand (Stefanics and Czigler, 2012), the contribution of the parvocellular activity seems to be necessary, therefore the fast identification indicated by the above studies holds for the processing of stimuli in vMMN studies.

## Data from steady-state evoked potential

In a passive paradigm (participants reacted to the color change of the fixation dot) Stothart et al. (2017) presented pictures of objects for 80 ms, with an 80 ms inter-stimulus interval. Every fifth picture (deviants) was from a different semantic category (e.g., non-living objects as deviants and animals as standards). According to the harmonic analysis, at the frequency of the deviant (1.25 Hz), the amplitude of the steady-state visual evoked potential increased, showing that using this RSVP procedure, 160 ms (from stimulus onset to stimulus onset) was enough for the implicit detection of a semantic category. Using related methods and facial stimuli Rossion and his colleagues obtained similar results (for a review see Rossion et al., 2020).

## Conclusions

Visual events violating sequential regularities elicit a modality-specific evoked potential (ERP) component, the visual mismatch negativity (vMMN) in the 120–350 ms latency range. Data from visual discrimination, identification, and steady-state evoked potential studies show that its latency is longer than the time needed to identify even complex visual events. Therefore, it is improbable that vMMN is a signature of the processes of early phases of stimulus identification. In the paradigms investigating vMMN (mainly in the passive oddball situation), events appear in the context of other ones. The context is the set of standard stimuli. Due to the regular appearance of standards, the system is capable of developing predictions about forthcoming events. I agree that the processes underlying vMMN are error signals, but signals of mismatch between expected and unexpected (unpredicted, surprising) *identified* events. These events have potential importance, even in a passive oddball paradigm. This suggestion is not new; it fits the early interpretation of (auditory) MMN by Risto Näätänen. “The mismatch negativity was interpreted by Näätänen et al. (1978) as reflecting a neural mismatch process, which represents an early, preattentive stage of change detection in the central nervous system and may, according to Näätänen (1979) lead to the elicitation of the orienting response…” (Lyttinen et al., 1992, p. 523). However, in case of an insignificant mismatch, no other components of the orientation reaction (activation and motor components) emerge (Lyttinen et al., 1992).

## Author contributions

IC: Conceptualization, Writing—original draft.

## Footnotes

^1^In this study the term visual perception (and perceptual) is considered as the whole set of processes from the activity of all structures that contribute to visual processing, i.e., both the lower and the higher level of the visual brain. In functional terms, it involves all processes leading to the identification of visual events, either with or without the involvement of consciousness.

^2^Recent studies apply the reverse control method. The deviant in a sequence is identical to the standard of the other sequence, and vice versa. This way physically identical stimuli are compared in the role of deviant and standard.
